# P15 Women’s satisfaction with the management of urinary tract infections during the COVID-19 pandemic: a qualitative analysis of free-text comments from a national survey in England

**DOI:** 10.1093/jacamr/dlad077.019

**Published:** 2023-08-02

**Authors:** A’mar Dababneh, Leigh Sanyaolu, Haroon Ahmed, Dushyanthi Alagiyawanna, Donna Lecky, Emily Cooper

**Affiliations:** Division of Population Medicine, Cardiff University, Cardiff, UK; Division of Population Medicine, Cardiff University, Cardiff, UK; Division of Population Medicine, Cardiff University, Cardiff, UK; Division of Population Medicine, Cardiff University, Cardiff, UK; Division of Population Medicine, Cardiff University, Cardiff, UK; Division of Population Medicine, Cardiff University, Cardiff, UK

## Abstract

**Background:**

Urinary tract infections (UTIs) are one of the most common bacterial infections affecting women. The COVID-19 pandemic altered how healthcare was accessed and delivered with rapid adoption of remote technologies. This study explored women’s satisfaction with the care they received for UTI in general practice during the pandemic.

**Methods:**

We analysed 799 responses to an open-ended question regarding women’s satisfaction with the management of their most recent UTIs. A thematic analysis was inductively conducted and supported by NVivo V.12.

**Results:**

Most women (72.7%) were satisfied with their UTI management. Main study themes related to consultation, management, patient’s feelings, and safety during the pandemic. Themes on positive aspects of care were related to thorough assessment and treatment by a doctor and having a positive experience with the healthcare provider (HCP). Themes on the negative aspects of care were related to long waiting times, use of virtual consultations, and patients feeling uninformed and invalidated about their UTIs.

**Conclusions:**

Patient satisfaction was closely linked to a positive face-to-face HCP interaction, prompt assessment and treatment, and discussion about prevention and self-care. To improve satisfaction, systems and consultations should account for patient choice in the HCP they consult with, allow for additional information about when and why a urine test should be provided, and ensure there is a platform for discussing patient preferences for antibiotic prescription or alternative therapy. Using resources to discuss these topics with patients may help address these issues, including risks and benefits of antibiotics, alternative treatments, prevention and self-care.

